# Optoelectronic synapse using monolayer MoS_2_ field effect transistors

**DOI:** 10.1038/s41598-020-78767-4

**Published:** 2020-12-14

**Authors:** Molla Manjurul Islam, Durjoy Dev, Adithi Krishnaprasad, Laurene Tetard, Tania Roy

**Affiliations:** 1 NanoScience Technology Center, University of Central Florida, Orlando, FL 32826 USA; 2 Department of Physics, University of Central Florida, Orlando, FL 32816 USA; 3 Department of Electrical and Computer Engineering, University of Central Florida, Orlando, FL 32816 USA; 4 Department of Materials Science and Engineering, University of Central Florida, Orlando, FL 32816 USA

**Keywords:** Electrical and electronic engineering, Electronics, photonics and device physics, Electronic devices

## Abstract

Optical data sensing, processing and visual memory are fundamental requirements for artificial intelligence and robotics with autonomous navigation. Traditionally, imaging has been kept separate from the pattern recognition circuitry. Optoelectronic synapses hold the special potential of integrating these two fields into a single layer, where a single device can record optical data, convert it into a conductance state and store it for learning and pattern recognition, similar to the optic nerve in human eye. In this work, the trapping and de-trapping of photogenerated carriers in the MoS_2_/SiO_2_ interface of a n-channel MoS_2_ transistor was employed to emulate the optoelectronic synapse characteristics. The monolayer MoS_2_ field effect transistor (FET) exhibits photo-induced short-term and long-term potentiation, electrically driven long-term depression, paired pulse facilitation (PPF), spike time dependent plasticity, which are necessary synaptic characteristics. Moreover, the device’s ability to retain its conductance state can be modulated by the gate voltage, making the device behave as a photodetector for positive gate voltages and an optoelectronic synapse at negative gate voltages.

## Introduction

The increasing demand for computational power and high cost of specific data transfer speeds between the memory and processors reveal the limitations of von Neumann architecture^1^. In traditional computing systems based on von Neumann architecture, memory and computational units are physically separated and connected by data bus. The bottleneck of von Neumann architecture arises from this data bus, which limits speed and efficiency and becomes inefficient while dealing with complex problems such as speech, image, and video data processing^2^. While neuromorphic systems can overcome this bottleneck by creating a network of artificial neurons and synapses, they do not respond directly to light stimulus. Bulky circuitry converts the image captured for pattern recognition to electrical signals which are then interpreted by the neuromorphic hardware. An optoelectronic synapse, similar to the sensory neurons in the human eye, can detect light and store information through conductance states, enabling optical sensing, storage and processing of data in the same device^3–5^. Arguably, the idea of optoelectronic synapse originated from the demonstration of optically tuned resistance states in poly(3-octylthiophene-2,5-diyl) (P3OT)-coated carbon nanotube transistors using the charge detrapping at the P3OT/gate dielectric interface^6^. Two dimensional (2D) materials established themselves as strong candidates for the realization of optoelectronic synapses, arising from their success in photodetection over a wide range of wavelengths^7^. Considerable efforts have been made in developing optoelectronic synapses with graphene, black phosphorus, and transition metal dichalcogenides due to their strong light-matter interactions and the ability to trap photoexcited carriers because of their large surface-to-volume ratio^1–5,8–15^. A photosensitive layer, such as perovskites to absorb light and a conductive channel to transport the photogenerated carriers, with the interface enabling charge trapping has been used to generate synaptic properties^11,13,14,16^. Alternatively, efficient charge trapping by engineering a floating gate^2^ or charge traps by functionalizing the channel/gate dielectric interface^17^ have enabled the demonstration of optoelectronic memory and synapses using MoS_2_. Synaptic behavior has been demonstrated in pristine films of amorphous oxide semiconductors, such as indium-gallium-zinc-oxide (IGZO) by tapping their inherent persistent photoconductivity^18^. Persistent photoconductivity, affected by gate voltage bias and measurement environment is known to be inherent in MoS_2_^19,20^. A monolayer MoS_2_/Si heterojunction diode exhibits properties of an optoelectronic synapse due to the persistent photoconductivity^19,20^. However, the use of this property to demonstrate optically-stimulated synaptic behavior in a monolayer MoS_2_ field effect transistor has not been explored yet.

In this work, we employ the persistent photoconductivity of monolayer MoS_2_ to mimic all necessary optical synapse characteristics such as short-term and long-term plasticity, photoconductance retention for at least 10^4^ s, paired pulse facilitation (PPF) and spike time dependent plasticity (STDP). The use of atomically thin monolayer MoS_2_ maximizes the charge trapping and de-trapping in the MoS_2_/gate oxide interface. This charge trapping ability can be modulated with an applied gate bias to operate the device as an optoelectronic synapse at a negative gate voltage and as a photodetector at zero or positive gate voltage. The mechanism of photogenerated charge trapping in the MoS_2_/gate oxide interface has been verified for both single crystal and polycrystalline monolayer MoS_2_ forming interface with either SiO_2_ or Al_2_O_3_ dielectric. We portray that the use of persistent photoconductivity of monolayer MoS_2_ as an optoelectronic synapse can constitute a paradigm shift to neuromorphic computing.

## Results and discussion

**Figure 1:**
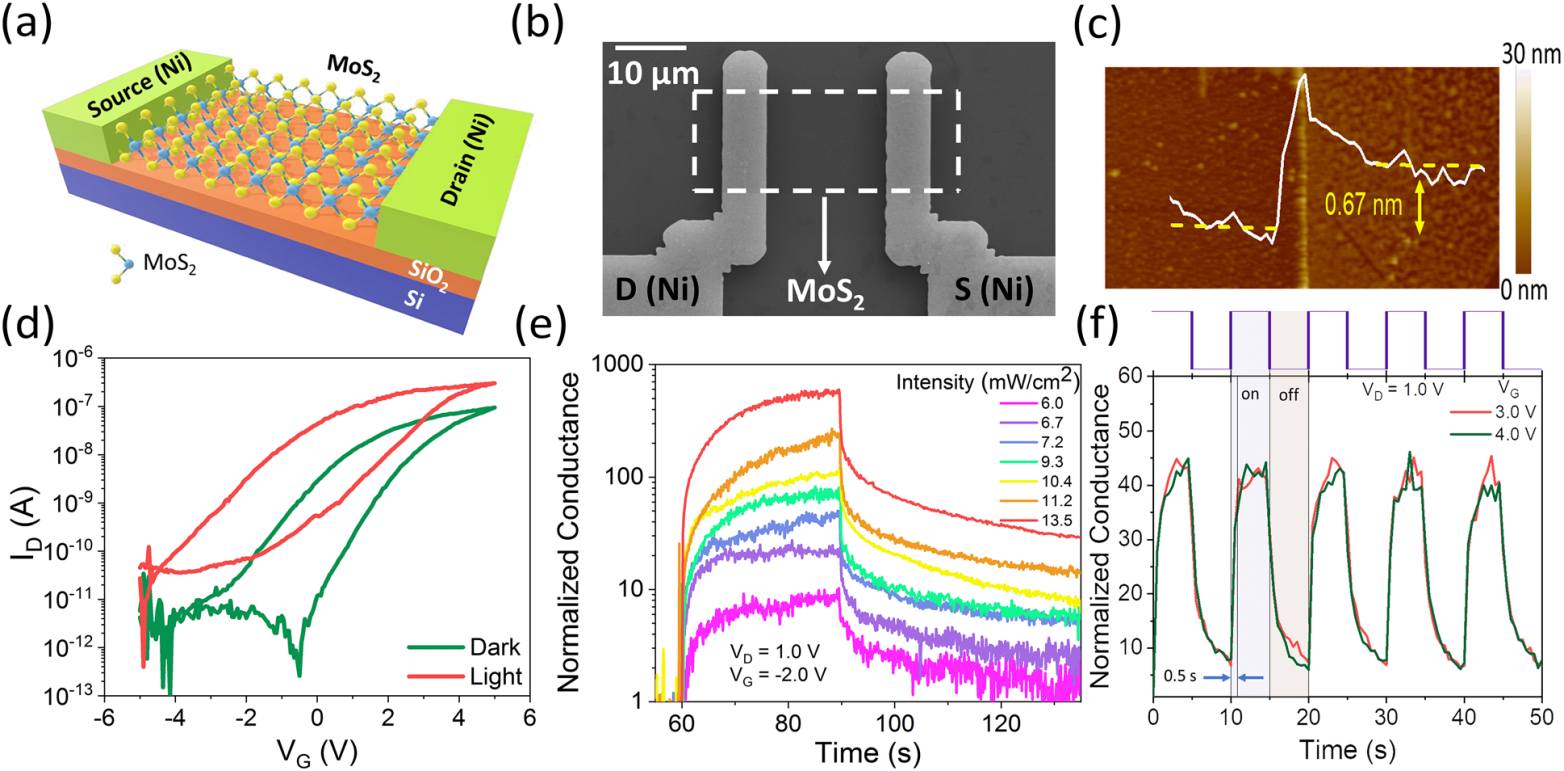
(**a**) Schematic diagram (not to scale) of back-gated monolayer MoS_2_ FET as optoelectronic synapse. (**b**) Representative SEM image of back-gated monolayer MoS_2_ FET. (**c**) AFM image of back-gated monolayer MoS_2_ FET. (**d**) *I*_*D*_*–V*_*G*_ at *V*_*D*_ = 1.0 V in dark and under light illumination (wavelength 450 nm and power 35.18 mW), showing the effect of photogenerated carriers. (**e**) Transient characteristics of the device showing change in the device conductance after applying a single light pulse (pulse duration 30 s) with varying intensity at *V*_*D*_ = 1.0 V and *V*_*G*_ = − 2.0 V. (**f**) Photo-switching characteristics of the monolayer MoS_2_ FET as photodetector at two different gate voltages under altering dark and light illumination.

Figure 1a depicts the schematic of a monolayer MoS_2_ FET used as an optoelectronic synapse. Monolayer MoS_2_ films were grown by CVD (Supplementary Figure S1a), as described in the Experimental section. Supplementary Figure S1b shows optical microscopic image of back gated monolayer MoS_2_ FET as optoelectronic synapse. Supplementary Figure S1c shows the Raman spectroscopy of the CVD-grown MoS_2_. *E*^1^_*2g*_ and *A*_*1g*_ modes of the MoS_2_ characteristic peaks are at 382.4 cm^−1^ and 400.3 cm^−1^, respectively. The energy difference between the two peaks is 17.9 cm^−1^ confirming the monolayer thickness of MoS_2_^8^. Supplementary Figure S1d shows the photoluminescence spectrum of the CVD-grown MoS_2_ film with a strong peak at 1.84 eV corresponding to the excitonic bandgap of the monolayer material^21^. Figure 1b shows the scanning electron microscope (SEM) image of a monolayer MoS_2_ FET fabricated on Si/SiO_2_ substrate. The thickness of the SiO_2_ layer is 10 nm. The device fabrication process is described in the Experimental section. Figure 1c shows the Atomic Force Microscopic (AFM) image of a monolayer MoS_2_ FET which confirms the thickness of monolayer MoS_2_^22,23^. Figure 1d shows the transfer characteristics of the MoS_2_ FET device with n-type behavior and on/off current ratio of 10^5^ in dark. Under light illumination of 450 nm wavelength, the photogenerated carriers cause a negative shift in threshold voltage and increases the drain current. The *I*_*D*_–*V*_*D*_ characteristics of the device in dark is shown in Supplementary Figure S2a. We examined the change in drain current enhancement for intensities of light varying from 6 to 13.5 mW/cm^2^ to confirm that the enhancement in drain current observed is indeed due to optical stimulus. Figure 1e shows the drain current normalized with respect to the dark current at *V*_*DS*_ = 1.0 V, *V*_*GS*_ = − 2.0 V as a function of time when the device is illuminated with a 450 nm light pulse for 30 s. The actual drain current values are shown in Supplementary Figure S2b. Supplementary Figure S2c shows the transfer characteristics at *V*_*D*_ = 1.0 V in dark and with different intensities of incident light. The threshold voltage shifts towards negative *V*_*G*_ with increasing intensities of incident light, which confirms the photogating effect of the device. The conductance of the device increases with increasing light intensity, which indicates accumulation of photogenerated carriers. The transient photo response of the monolayer MoS_2_ FET device is shown in Fig. 1f when a train of light pulses is applied to the device keeping the gate voltage positive and drain voltage at 1.0 V. The device exhibits a quick rise to a high conductance state in a repeatable manner with the application of lightand reverts to its initial low conductance state upon removal of light, indicating that the device can be used as a light-activated switch. The response time for the device conductance to increase to 80% is approximately 0.5 s. The photocurrent decay time is also similar for 80% decay from its maximum value. No obvious change in the rise and fall times are observed for the different positive gate voltages applied. The device exhibits a photoresponsivity *R* of 12.03 AW^−1^ and a detectivity D* of 4.0 × 10^9^ Jones. The photoresponsivity is calculated according to the equation *R* = *I*_*ph*_*/P*_*inc*_*A*, where, *I*_*ph*_ is the photocurrent given by *I*_*light *_*– I*_*dark*_, *P*_*inc*_ is the power of incident light and *A* is the area of the photoactive channel. *P*_*inc*_ was 35.18 mW, where V_D_ and V_G_ was kept at 1.0 V and 3.0 V, respectively. The detectivity is calculated as D* = *RA*^*0.5*^*/*(*2eI*_*dark*_)^*0.5*^, where, *R* is the responsivity, *A* is the area of the photoactive channel and *e* is the absolute value of electronic charge. These values are consistent with previously reported results on MoS_2_ phototransistors^24–26^.

**Figure 2:**
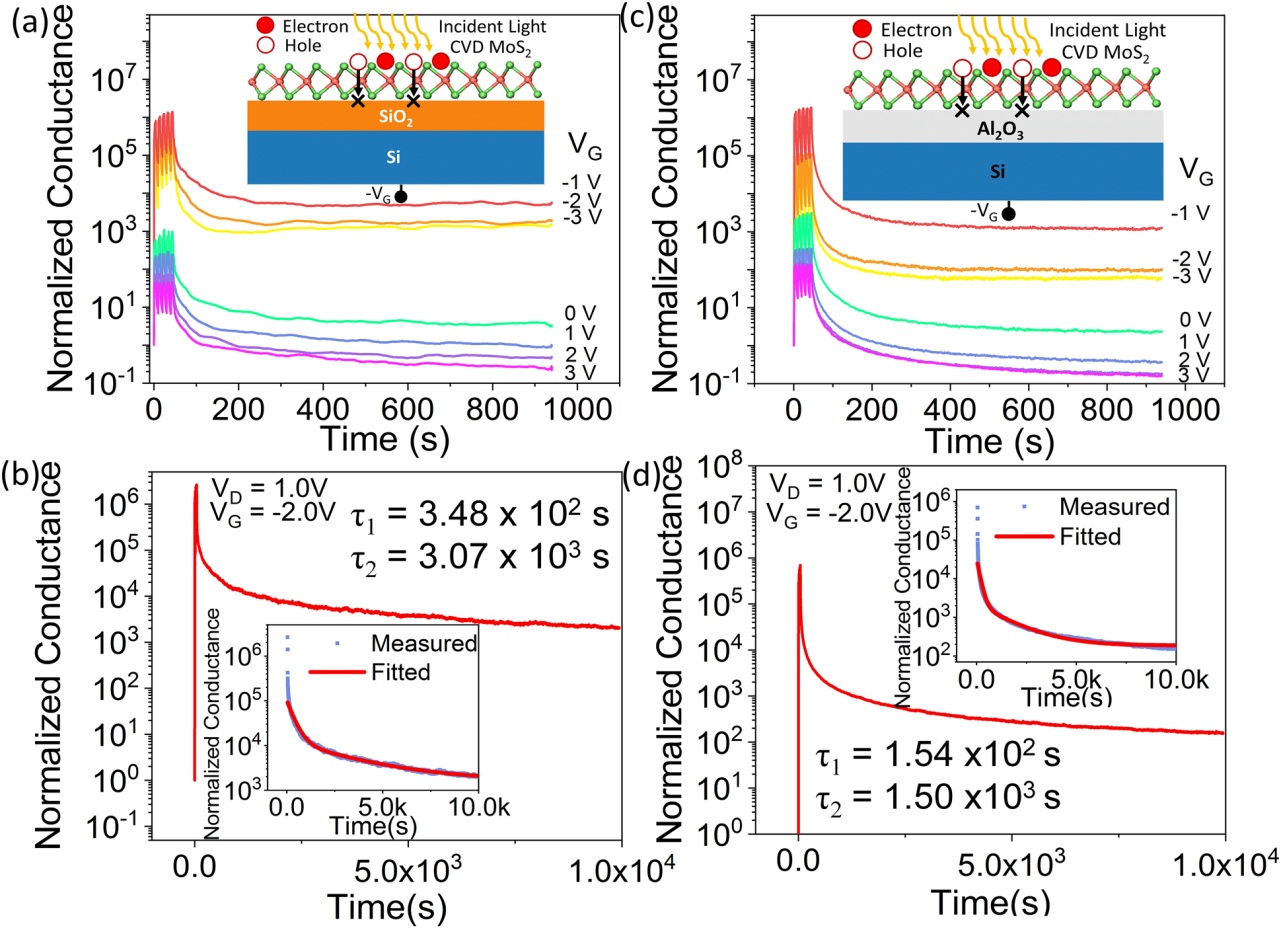
CVD grown monolayer MoS_2_ on SiO_2_ gate: (**a**) Gate-tunable potentiation and conductance retention. (**b**) Retention for 10^4^ s, after applying 5 light pulses. Inset shows the retention curve fitted with an exponential decay function. CVD grown monolayer MoS_2_ on Al_2_O_3_ gate: (**c**) Gate-tunable potentiation and conductance retention. (**d**) Retention for 10^4^ s, after applying 5 light pulses (5 s on/5 s off). Inset shows the retention curve fitted with an exponential decay function.

We further examined the effect of negative gate voltages on the device characteristics, since prior reports show that the maximum responsivity of MoS_2_-based phototransistors occur at negative gate voltages^20^. We applied a train of 5 light pulses with on and off time of 5 s each, for a constant *V*_*D*_ of 1.0 V and for gate voltages varying from − 3.0 to 3 V. The photocurrent was recorded continuously up to 1000 s. The light stimulated potentiation and drain current retention of CVD grown monolayer MoS_2_ on SiO_2_ gate at different gate voltages are shown in Supplementary Figure S3. The device conductance normalized to the dark current is shown in Fig. 2a for gate voltages varying from − 1 to 3 V. For *V*_*G*_ ≥ 0 V, the device behaves as a photodetector, as described earlier, with the photocurrent decaying quickly to the initial dark current level when the light is turned off. However, for *V*_*G*_ < 0 V, we observed that the device conductance increases for each incident light pulse, resembling the potentiation of a synapse. This conductance is hereby referred to as the post synaptic current. When the light is withdrawn, the device does not return to its initial dark current state for a long period of time. The level of conductance that the device maintains after the light pulses are withdrawn is determined by the negative gate voltage applied. At negative gate bias, the photogenerated holes are injected towards the gate oxide and get trapped in the SiO_2_/MoS_2_ interface trap centers (inset of Fig. 2a). Thus, the photogenerated electrons are unable to recombine with the trapped holes and contribute to the high conductance of the n-channel MoS_2_ device. As the gate voltage is increased from − 3.0 to − 1.0 V with the train of light pulses being applied, the photocurrent increases due to photogating effect. The normalized device conductance increases when the light pulses are withdrawn with decreasing negative *V*_*G*_ with increasing number of photogenerated electrons as the holes get trapped at the SiO_2_/MoS_2_ interface. This high normalized conductance maintained after the light is withdrawn is referred to as the retention of the conductance state. But, when *V*_*G*_ ≥ 0 V is applied, despite the larger number of photogenerated electrons and holes, the absence of a negative gate field keeps the carriers together in the channel causing them to recombine soon after the light is withdrawn. The device does not retain its photoconductance in dark for *V*_*G*_ ≥ 0 V. Figure 2b shows the conductance retention of CVD grown monolayer MoS_2_ on 10 nm SiO_2_ gate oxide after the application of five light pulses to potentiate the device, at *V*_*D*_ = 1.0 V and *V*_*G*_ = − 2.0 V. The device retains its conductance state for at least 10^4^ s after the light pulses are withdrawn. The normalized conductance retained in dark decays with time following a double exponential function *Y* = *1* + *C*_*1*_* exp *(*− t*/*τ*_*1*_) + *C*_*2*_* exp *(*− t/τ*_*2*_), which is plotted in the inset of Fig. 2b. Here, *t* is the time, *C*_*1*_, *C*_*2*_ are initial conductance magnitudes and *τ*_*1*_, *τ*_*2*_ are the fast and slow decay time constants, respectively. We obtain a small time constant *τ*_*1*_ = 3.48 × 10^2^ s, symbolizing a rapid relaxation to an intermediate conductance state after light withdrawal and a larger time constant τ_2_ = 3.07 × 10^3^ s indicating the long term potentiation (LTP) of the device. Thereby, LTP was induced in the device by applying 5 light pulses and it was sustained for 10^4^ s.

In order to explore the role of the gate dielectric/channel interface on photogenerated hole-trapping and conductance retention, we sought to alter the gate dielectric from thermally grown SiO_2_ to atomic layer deposited Al_2_O_3_. CVD grown monolayer MoS_2_ was used as the n-channel on 20 nm thick Al_2_O_3_ gate dielectric to fabricate the FET device and the same measurements were repeated as shown in Fig. 2a,b. Figure 2c represents the light induced potentiation and conductance retention for varying gate voltages. The device behavior is similar to when SiO_2_ is used as the gate dielectric. The actual drain currents as a function of time for varying *V*_*G*_ are shown in Supplementary Figure S4. The device retains its photoconductance for 10^4^ s at *V*_*G*_ < 0 V, as shown in Fig. 2d. The inset of Fig. 2d shows the post-synaptic normalized conductance fitted with the previously used double exponential function, to obtain the fast and slow decay time constants of *τ*_*1*_ = 1.54 × 10^2^ s and *τ*_*2*_ = 1.50 × 10^3^ s, respectively, which are in the same range as in the case of SiO_2_ gate dielectric.

**Figure 3:**
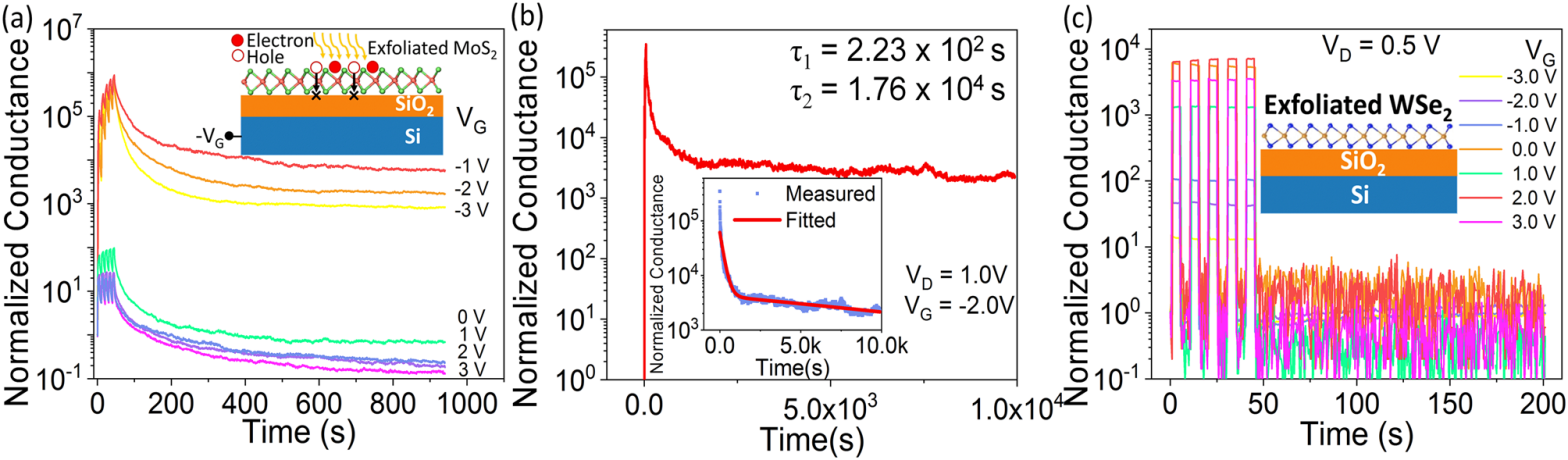
Exfoliated monolayer MoS_2_ on SiO_2_ gate: (**a**) Gate-tunable potentiation and conductance retention, (**b**) Retention for 10^4^ s, after applying 5 light pulses. Inset shows retention curve fitted with an exponential decay function. (**c**) Potentiation and zero conductance retention of exfoliated WSe_2_ on SiO_2_ gate at different gate voltages.

Memristive behavior in CVD-grown monolayer MoS_2_ has been attributed to carrier trapping at grain boundaries^27^. Now, to examine the possibilities of the photogenerated carriers getting trapped in the grain boundaries of MoS_2_, the same measurements were repeated with exfoliated monolayer MoS_2_ as the n-channel of the FET on SiO_2_ gate dielectric. Figure 3a shows the gate tunable potentiation and retention for single-crystal exfoliated MoS_2_ flake, similar to the polycrystalline CVD-grown MoS_2_ film. The characteristics in terms of drain current is shown in Supplementary Figure S5. At a negative gate bias, the exfoliated MoS_2_ device retains its high photoconductance state for at least 10^4^ s, as presented in Fig. 3b. The inset of Fig. 3b shows the normalized photoconductance fitted with the double exponential function with fast and slow decay time constants of *τ*_*1*_ = 2.23 × 10^2^ s and *τ*_*2*_ = 1.76 × 10^4^ s, respectively. While the fast time constant is in the same range as the CVD-grown MoS_2_ cases, the slow time constant is an order of magnitude higher for the exfoliated MoS_2_ case, indicating the role of grain boundaries in reducing the persistent photoconductivity of MoS_2_. However, it is worth noting that both single crystal (exfoliated) and polycrystalline (CVD) monolayer MoS_2_ forming interface with either SiO_2_ or Al_2_O_3_ dielectric can trap photogenerated holes substantially. An interesting question is whether other 2D materials would show similar photoconductance retention. To seek the answer, we fabricated FET devices using exfoliated WSe_2_ as the channel material (Supplementary Figure S6a) and conducted the same measurements as shown in Fig. 3a. The WSe_2_ device behaves as a p-type semiconductor as shown in the *I*_*D*_*–V*_*G*_ and *I*_*D*_*–V*_*D*_ (dark) in Supplementary Figure S6b,c. Figure 3c shows that the WSe_2_ device conductance does not keep increasing with each light pulse applied for any gate voltage in the range of − 3 V to + 3 V, in contrast with MoS_2_ devices. The characteristics in terms of actual drain current is shown in Supplementary Figure S6. Thus, we confirm that the persistent photoconductivity is inherent in monolayer MoS_2_ and can be used to demonstrate the functionalities of an optoelectronic synapse.

**Figure 4:**
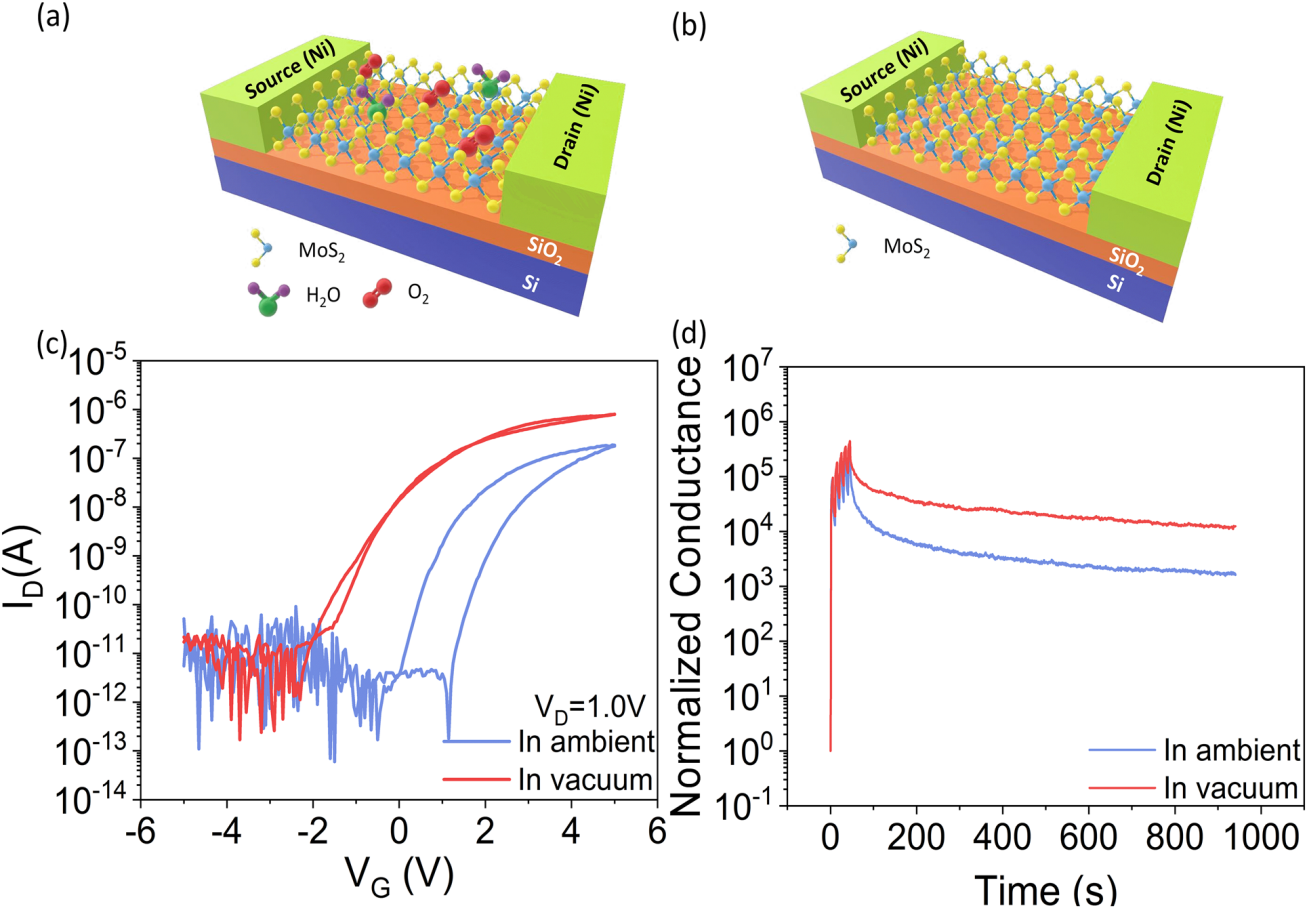
Schematic of CVD grown monolayer MoS_2_ FET in (**a**) ambient and (**b**) vacuum condition. (**c**) Comparison of *I*_*D*_*–V*_*G*_ at *V*_*D*_ = 1.0 V in ambient and in vacuum. (**d**) Comparison of potentiation and conductance retention at *V*_*D*_ = 1.0 V and *V*_*G*_ = *V*_*Threshold*_ – 0.5 V of MoS_2_ FET in ambient and in vacuum.

To establish the role of device environment in the transfer characteristics and the transient photocurrent characteristics, we measured the CVD-grown monolayer MoS_2_ devices on SiO_2_ gate dielectric both in ambient air and in vacuum. In ambient air, the MoS_2_ channel material absorbs O_2_ and H_2_O molecules from the environment, which causes hysteresis in the transfer characteristics^19,28–30^. The schematics of the device in ambient and in vacuum are depicted in Fig. 4a,b, respectively. The *I*_*D*_*–V*_*G*_ of the device in ambient and in vacuum is presented in Fig. 4c. It shows that the hysteresis in transfer characteristics decreases drastically in vacuum and the threshold voltage of the device shifts negatively from − 0.5 to − 2.5 V. The drain current also increases by an order of magnitude in vacuum, consistent with previous reports^19^. After the application of 5 light pulses, the normalized photoconductance retention of the same device in ambient and in vacuum at gate voltage *V*_*G*_ = *V*_*threshold *_– 0.5 V (in ambient, V_G_ = − 1.0 V and in vacuum, V_G_ = − 3.0 V) are shown in Fig. 4d. The retention of the device also increases by 10 times in vacuum when compared to the case in ambient. These observations suggest that, in ambient condition, some electrons are trapped in the absorbed O_2_ and H_2_O molecules on the MoS_2_ surface resulting in a decreased drain current in air^19^. Thereby, removal of those absorbed O_2_ and H_2_O molecules in vacuum enhances the conductance and the photoconductance retention of the device. This also proves that the high photoconductance retention of the device observed at negative gate voltages is not due to the absorbed O_2_ and H_2_O molecules but due to defects at the gate dielectric/MoS_2_ interface.

**Figure 5:**
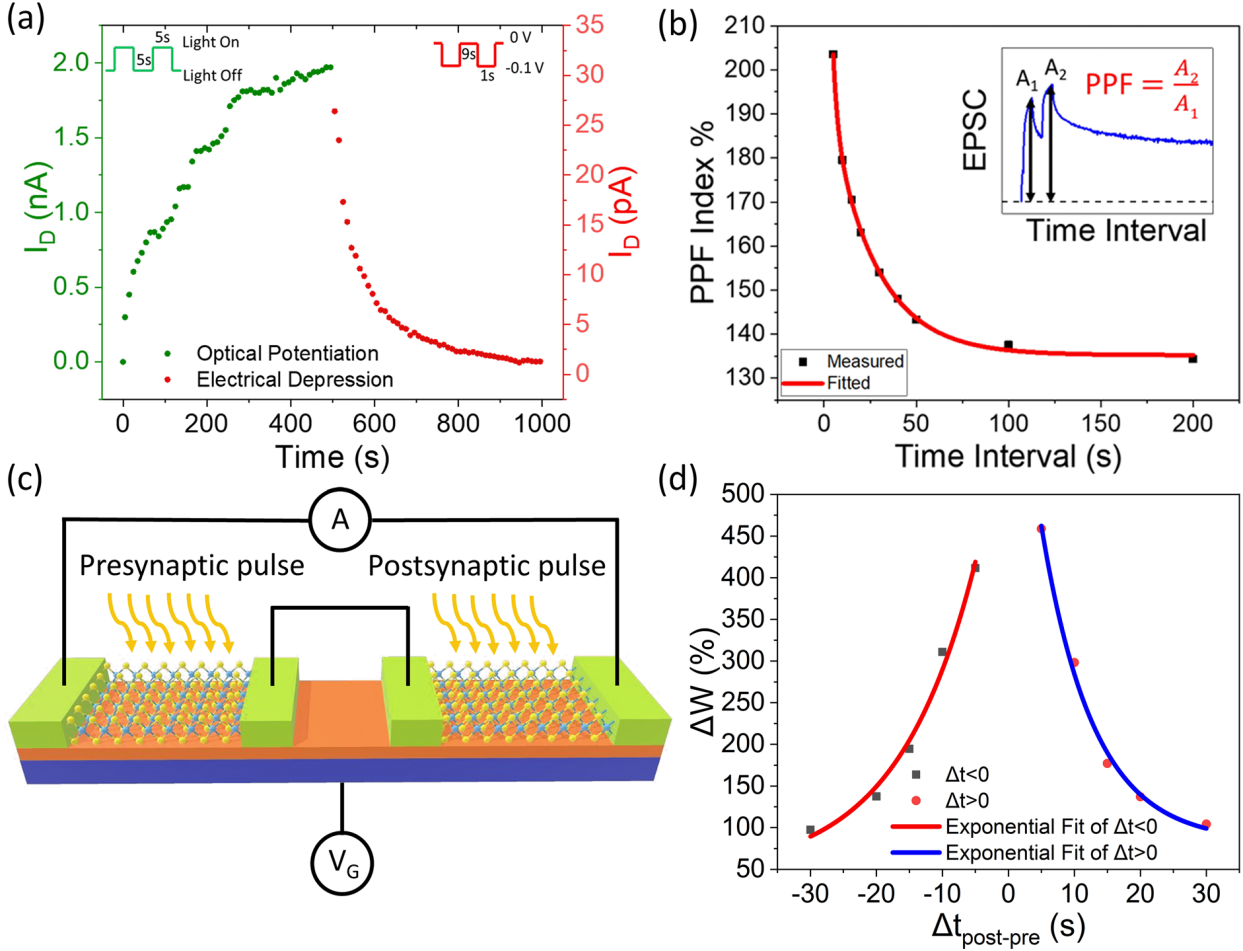
MoS_2_ FET as optoelectronic synapse. (**a**) Optical potentiation by applying 50 light pulses (5 s on/5 s off) at *V*_*D*_ = 1.0 V and electrical depression by applying 50 electrical pulses at the drain of amplitude − 0.1 V and duration 1 s in dark, *V*_*G*_ = − 2 V is maintained throughout. (**b**) Excitatory post-synaptic current induced PPF index of the optoelectronic device with respect to time interval between two consecutive pulses measured at *V*_*D*_ = 1.0 V and *V*_*G*_ = − 2.0 V. Inset shows transient photocurrent of the device for applying two consecutive light pulses. (**c**) A schematic showing two connected optoelectronic synaptic devices for the emulation of spike-timing-dependent plasticity and (**d**) Emulation of spike time dependent plasticity.

With the understanding of persistent photoconductivity of MoS_2_, we now demonstrate an optoelectronic synapse using the simple CVD-grown monolayer MoS_2_ FET. Long-term plasticity and short-term plasticity of biological synapses are two cooperative operations to complete learning and memory functionalities in human brain^31^. Long-term plasticity enables neuromorphic synaptic devices to hold the memory for a long period of time while short-term plasticity enables the device to relax back to its initial low conductance state^3^. To emulate these conditions, our MoS_2_ device on SiO_2_ gate dielectric was potentiated by applying light pulses and depressed by applying electrical pulses at the drain. Figure 5a shows the optical potentiation and electrical depression of the optoelectronic synapse device. The device was potentiated by applying 50 light pulses (on and off time of 5 s each) at constant drain bias of 1.0 V, *V*_*G*_ = − 2 V. The device was depressed by applying 50 electrical pulses (on time 1 s) of amplitude − 0.1 V at the drain, with *V*_*G*_ = − 2 V. The non-linearity factor extracted from potentiation and depression is 2.92 and 5.49, respectively and the asymmetry is 2.57^15^. This represents the conductance tuning required for training of the optoelectronic synapse for neural network applications. The negative voltage pulses applied at the drain de-traps the holes from the SiO_2_/MoS_2_ interface and thus gradually depress the device to its initial low conductance state. The optoelectronic synapse device follows paired pulse facilitation (PPF), an important synaptic learning rule involving short term plasticity of the synapse, as represented in Fig. 5b. The increase in device conductance for the application of light pulse corresponds to the excitatory post-synaptic current (EPSC). The PPF index, defined as the ratio of the amplitudes between the second EPSC (*A*_*2*_) and the first EPSC (*A*_*1*_), is plotted as a function of time interval *Δt*. The inset of Fig. 5b shows the transient photocurrent upon application of two successive pulses. PPF signifies the fact that the second light pulse results in a larger post-synaptic current compared to the first pulse when two consecutive pulses are applied to the device^8^. The PPF index is high for a small interval between two applied light pulses as the photogenerated carriers from the first light pulse combine with photogenerated carriers from the second light pulse before recombination, resulting in an enhancement in the conductance state of the device. With increasing time interval between successive light pulses, the PPF index decreases gradually. PPF index is found to decay with *Δt* following a double exponential function: Y = *1* + *D*_*1*_* exp *(*−* *Δt/τ*_*1*_) + *D*_*2*_* exp *(*−* *Δt/τ*_*2*_), where, Y = *A*_*2*_*/A*_*1*_*, Δt* is the time interval between two consecutive pulses, *D*_*1*_, *D*_*2*_ are the initial facilitation magnitudes and *τ*_*1*_, *τ*_*2*_ are the characteristic relaxation time of the rapid and slow decay term, respectively. The relaxation time obtained are *τ*_*1*_ = 2.45 s and *τ*_*2*_ = 24.78 s, which are consistent with those of biological synapses^8^. Our neuromorphic synaptic device shows a maximum PPF index of 203.5% for a time interval of 5 s between two consecutive light pulse. Supplementary Figure S7 shows the change in photocurrent with varying time interval between two consecutive light pulses, each pulse having a duration of 5 s. In neuromorphic operation, the intercoupling between pre-synaptic and post-synaptic activities is defined by spike time dependent plasticity (STDP), which is measured by the change in synaptic weight due to relative timing between them^32–34^. To mimic the STDP function, two separate devices were used, and the source contact of one device was electrically shorted with the drain contact of the other device. The schematic diagram of the connected devices is depicted in Fig. 5c. The light pulses on the first and second device are referred as pre-synaptic and post-synaptic pulse, respectively. The governing parameter which determines the connection strength is the time interval between the light pulses, *Δt*_*post-pre*_ = *t*_*post*_ *–* *t*_*pre*,_ where, *t*_*post*_ is the time when post-synaptic pulse is turned off and *t*_*pre*_ is the time when pre-synaptic pulse is turned on. The STDP induced change in synaptic weight (*ΔW*) between the pre-synaptic and post-synaptic device is calculated as: *ΔW* = (*I*_*post*_ *–* *I*_*pre*_)/*I*_*pre*_. Here, *I*_*post*_ and *I*_*pre*_ are the currents induced by post-synaptic and pre-synaptic pulse, respectively^18^. Figure 5d shows the change in synaptic weight as a function of time interval *Δt*_*post–pre*_. For *Δt* > 0, light pulse (on time 5 s) was applied on pre-synaptic device first and then the post-synaptic device at *V*_*G*_ = − 2.0 V and *V*_*D*_ = 1.0 V. On the other hand, for *Δt* < 0, light pulse (on time 5 s) was applied on post-synaptic device first and then the pre-synaptic device at the same *V*_*G*_ and *V*_*D*_. Exponential growth function: Y = *1* + *F*_*1*_* exp *(*R*_*0*_*Δt*) was used to fit for *Δt* < 0 and exponential decay function Y = *1* + *F*_*2*_* exp *(*−* *Δt/τ*) was used to fit for *Δt* > 0, where, *R*_*0*_ is the initial rate of growth, *Δt* is the time interval between two applied pulses and *τ* is the characteristic relaxation time. A symmetric STDP characteristic was observed for varying *Δt*_*post–pre*_ from − 30 to + 30 s. The change in synaptic weight was maximum for small |*Δt*_*post–pre*_| and it decreased exponentially with increasing |*Δt*_*post–pre*_|. Supplementary Figure S8 shows the transient photocurrent characteristics with varying time intervals between the pre-synaptic and post-synaptic pulse.

## Conclusion

An optoelectronic synapse is realized using monolayer MoS_2_ as a conducting channel by a simple strategy of SiO_2_/MoS_2_ interfacial charge trapping causing persistent photoconductivity in MoS_2_. The strategy is based on injecting photogenerated holes towards the SiO_2_/MoS_2_ interface by applying a negative gate voltage. The trapping and de-trapping of photogenerated holes at the SiO_2_/MoS_2_ interface enables the monolayer MoS_2_ FETs to emulate all necessary optical synapse characteristics such as short-term and long-term plasticity, photoconductance retention for at least 10^4^ s, PPF and STDP. Moreover, the monolayer MoS_2_ device is gate tunable to behave as a photodetector with no conductance retention at *V*_*G*_ ≥ 0 V. Emulating a biological optical synapse by a simple monolayer MoS_2_ FET utilizing the gate dielectric/channel interface charge trapping mechanism opens up new possibilities for machine vision technologies for artificial intelligence.

## Methods

### Materials and device fabrication

For CVD growth, MoS_2_ powder precursor of molecular weight 160.07 was purchased from Sigma Aldrich. High quality and large area of monolayer MoS_2_ was synthesized directly on 2.0 × 2.0 cm^2^ Si/SiO_2_ substrate by Chemical Vapor Deposition (CVD) method. MoS_2_ powder was used as precursor and was placed upstream at the center of the heating zone. The substrate was placed downstream at a distance 7.0 cm from the precursor. 1553 sccm flow of Ar was used as the carrier gas. 950 °C temperature was applied to the precursor and the substrate was approximately at 700 °C. For a growth time of 30 min, Si/SiO_2_ substrate was completely covered with a monolayer film of MoS_2_. The CVD grown monolayer MoS_2_ was coated with a thin layer of poly (methyl methacrylate) (PMMA), followed by drying at room temperature for 12 h. The PMMA coated sample was then floated on buffer oxide etch (BOE, 40% NH_4_F/49% HF, 6:1 v/v in water) for 12 h to remove the SiO_2_ under the MoS_2_ film. The MoS_2_ film was released from the substrate and held together by the PMMA supporting layer, floated on the acid bath. The floating MoS_2_ film with PMMA was then transferred to a deionized water bath and kept floating for 12 h to remove the acid remnant. The source/drain contacts were patterned on a separate SiO_2_ (10 nm)/Si substrate followed by e-beam evaporation of Ni (60 nm). The MoS_2_ film with PMMA was then transferred on the target SiO_2_ (10 nm)/Si substrate and dried for 15 min. The sample was heated on a hot plate at 150 °C for 5 min and the PMMA layer was removed by immersing the sample in acetone for 3 h. The MoS_2_ film was patterned by photolithography and etched in O_2_ plasma.

### Device characterization

Raman spectroscopy was performed on a Renishaw RM 1000B Micro-Raman Spectrometer with excitation of 514 nm. The PL spectra was recorded using WITec Alpha300 with excitation of 532 nm and integration time of 10 s. We performed electrical characterization with HP 4156A precision semiconductor parameter analyzer on a Janis cryogenic probe station in air and in vacuum at a pressure of 10^–4^ Torr. A 450 nm laser was used as the light source. Light intensity was measured by Daystar's DS-05A meter. All electrical measurements were conducted at room temperature.

## Supplementary Information

Supplementary Figures.

## Data Availability

All data generated and analyzed during this study are either included in the published article itself (or available within the Supplementary Information files).
