# Supplementary material for: Optoelectronic synapse using monolayer MoS2 field effect transistors
Source: Sci Rep. 2020 Dec 14;10:21870. doi: 10.1038/s41598-020-78767-4 (PMC7736870; doi:10.1038/s41598-020-78767-4)
Supplement: Supplementary file 1 — Supplementary Figures. [file 41598_2020_78767_MOESM1_ESM.pdf]

Optoelectronic Synapse Using Monolayer MoS_2 Field Effect Transistors

*Molla Manjurul Islam,[1, 2] Durjoy Dev,[1, 3] Adithi Krishnaprasad,[1, 3] Laurene Tetard, [1, 2] and Tania Roy[1, 2, 3, 4, *]*

[1]NanoScience Technology Center, University of Central Florida, Orlando FL 32826, USA

[2]Department of Physics, University of Central Florida, Orlando FL 32816, USA

[3]Department of Electrical and Computer Engineering, University of Central Florida, Orlando FL 32816, USA

[4]Department of Materials Science and Engineering, University of Central Florida, Orlando FL 32816, USA

[*]Corresponding author: tania.roy@ucf.edu

Supporting Information

S1. CVD growth of large area monolayer MoS_2, material characterization and mobility calculation.

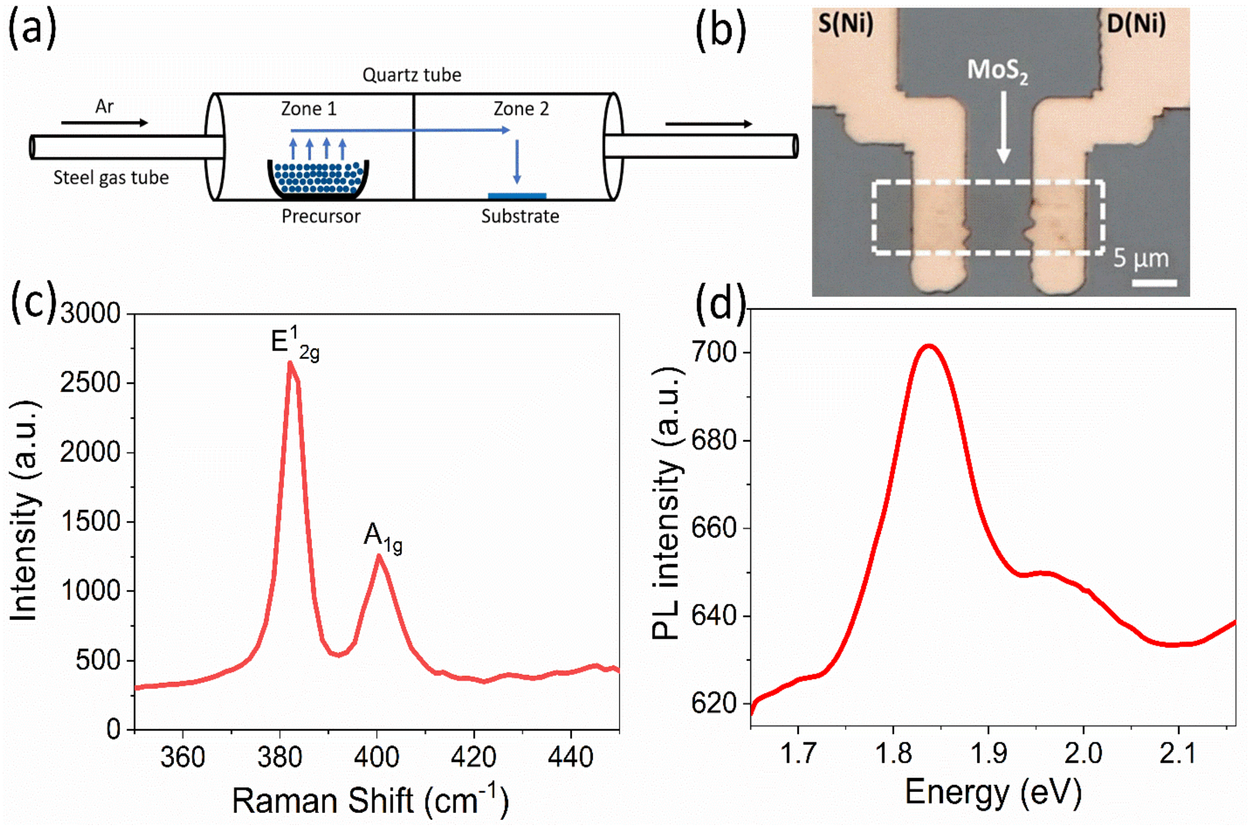

Figure S1. (a) Schematic (not to scale) representation of chemical vapor deposition of monolayer MoS_2. (b) Optical microscopic image of back gated monolayer MoS_2 FET as optoelectronic synapse (c) Raman and (d) PL spectrum of CVD grown monolayer MoS_2.

The typical field effect mobility of the monolayer MoS_2 FET in vacuum is 1.25 $cm^2\ V^{-1}\ s^{-1}$, calculated as per the equation $\mu_{FE} = (dI_D/dV_G)\ (L/W)\ (1/C_{ox}V_D)$, where μ_{FE} is the field-effect mobility, C_{ox} is the capacitance of the gate oxide, L is the length and W is the width of the conductive channel.

S2. CVD-grown MoS_2 on SiO_2 gate.

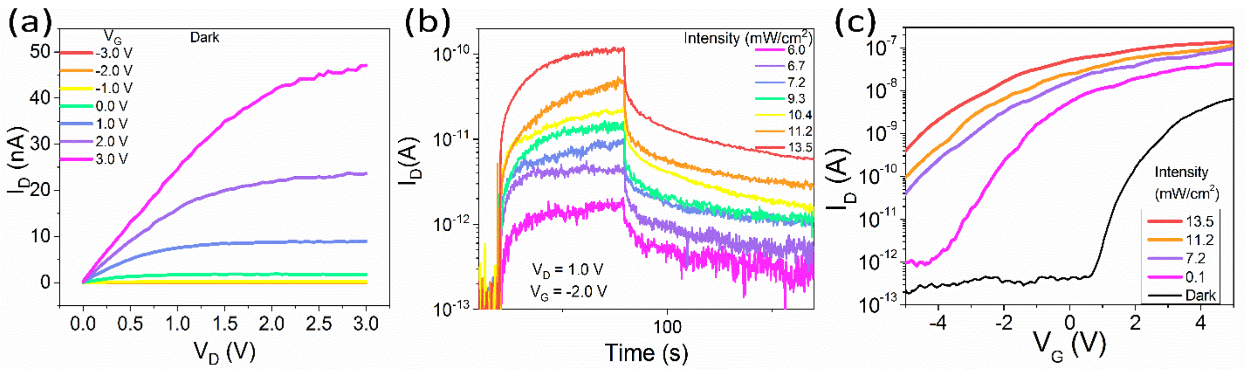

Figure S2. (a) I_D - V_D of CVD grown MoS_2 FET as Optoelectronic Synapse on SiO_2 gate at different V_G in dark. (b) Transient characteristics of the device showing change in the drain current after applying a single light pulse (pulse duration 30 s) with varying intensity at V_D = 1.0 V and V_G = -2.0 V. (c) I_D - V_G at V_D = 1.0 V in dark and with different intensities of incident light.

Figure S2a shows I_D - V_D of CVD grown MoS_2 FET as Optoelectronic Synapse on SiO_2 gate at different V_G in dark. With increasing V_D, the drain current, I_D also increases linearly. The measurement was carried out for gate voltages starting from -3.0 V to 3.0 V. **Figure S2b** represents the transient characteristics of the device showing change in the drain current after applying a single light pulse (on time 30 s) with varying intensity at V_D = 1.0 V and V_G = -2.0 V. With increasing intensity, drain current, I_D also increases. It signifies the increase in photogenerated carriers. **Figure S2c** shows the I_D - V_G at V_D = 1.0 V in dark and with different intensities of incident light, which confirms the photogating effect of the device.

S3. Drain current retention of CVD grown MoS_2 on SiO_2 gate.

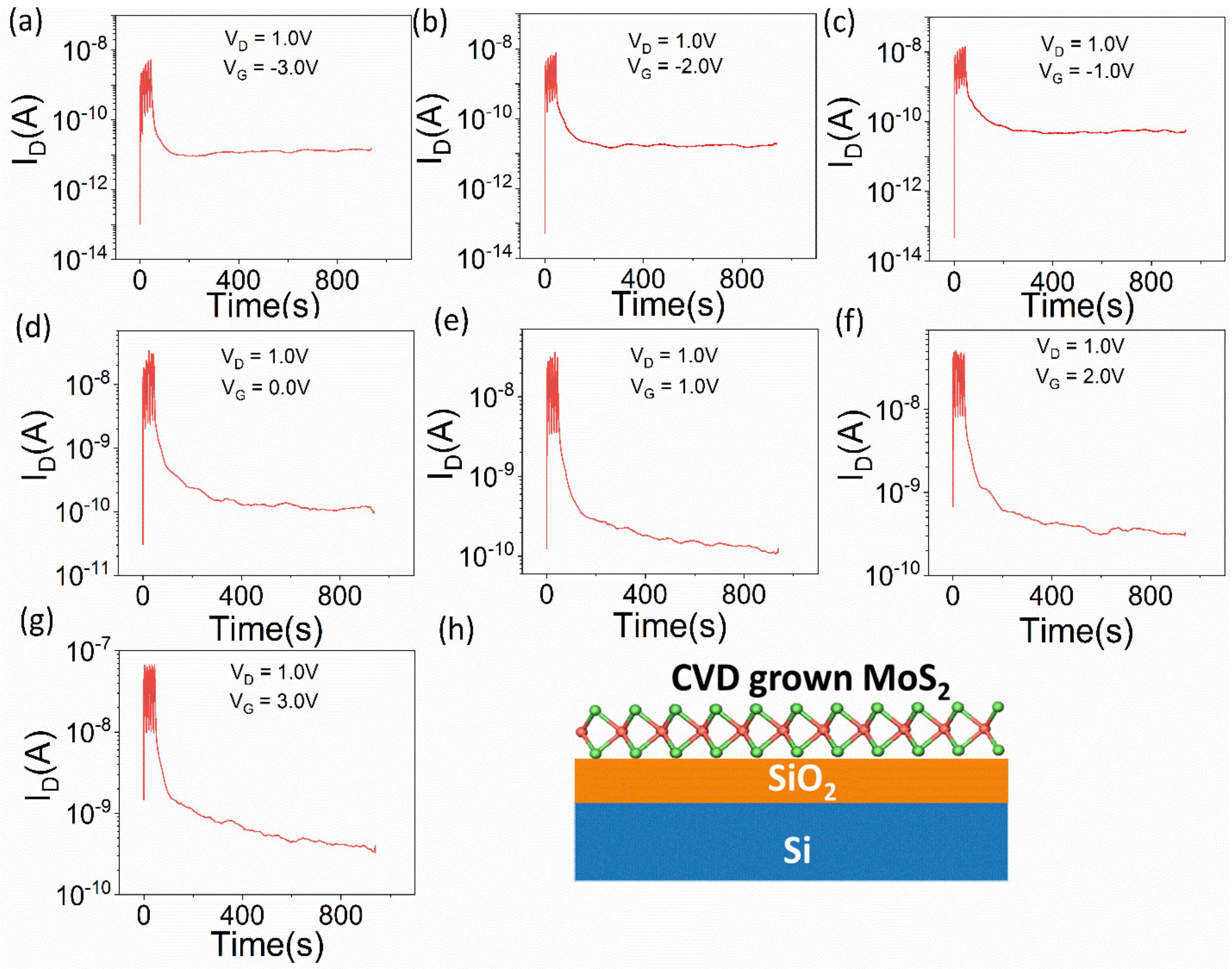

Figure S3. Light stimulated potentiation and drain current retention of CVD grown monolayer MoS_2 on SiO_2 gate at (a) V_G = -3.0 V. (b) V_G = -2.0 V. (c) V_G = -1.0 V. (d) V_G = 0.0 V. (e) V_G = 1.0 V. (f) V_G = 2.0 V. (g) V_G = 3.0 V. (h) Schematic diagram of Si/SiO_2/CVD grown MoS_2.

Figure S3 shows the light stimulated potentiation and drain current retention of CVD grown monolayer MoS_2 on SiO_2 gate at different gate voltages. At negative gate voltages, device potentiates with application of each light pulse and retains a huge part of drain current after removing the light source. Device shows maximum retention at V_G = -1.0 V.

S4. Drain current retention of CVD grown MoS_2 on Al_2O_3 gate.

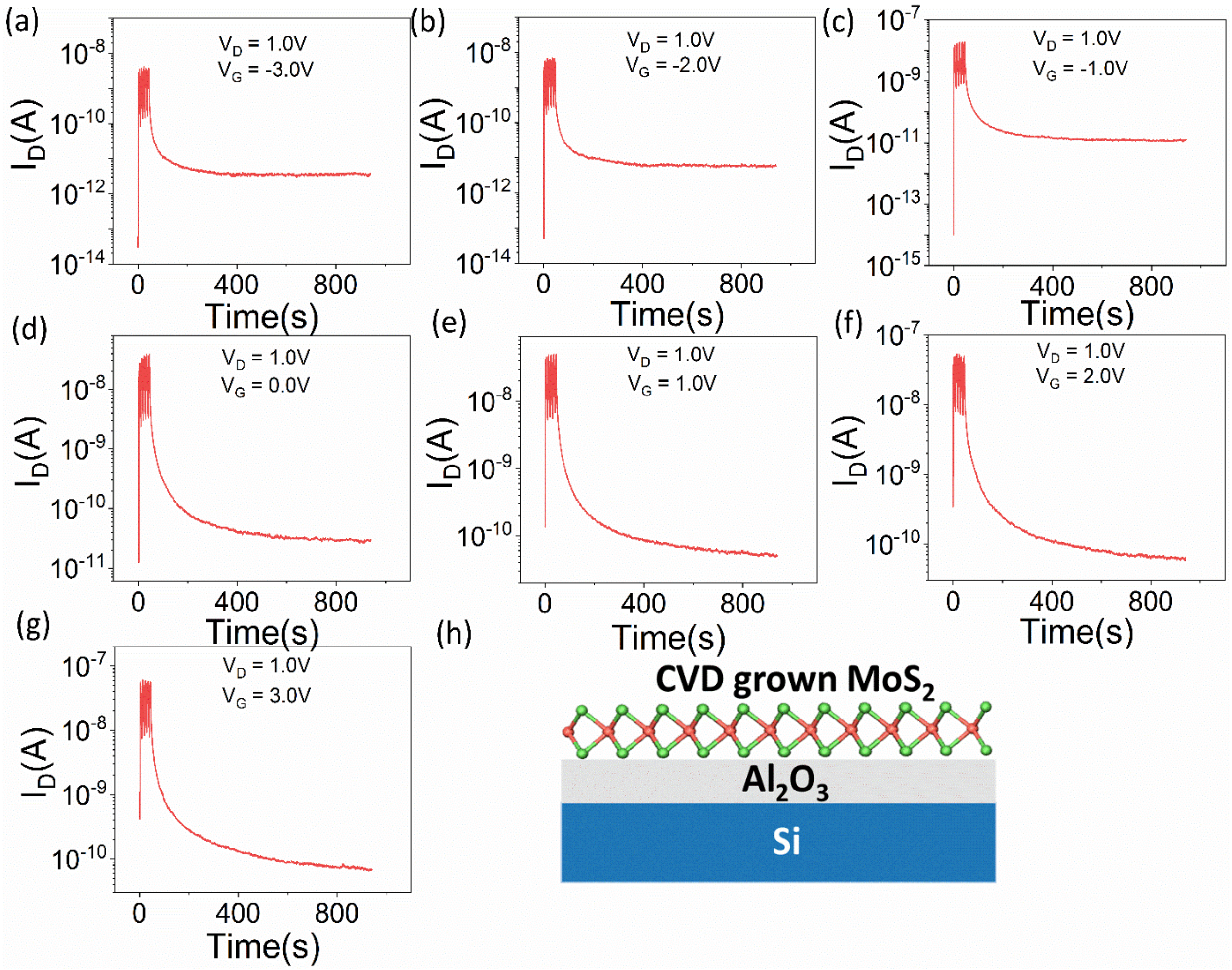

Figure S4. Light stimulated potentiation and drain current retention of CVD grown monolayer MoS_2 on Al_2O_3 gate at (a) V_G = -3.0 V. (b) V_G = -2.0 V. (c) V_G = -1.0 V. (d) V_G = 0.0 V. (e) V_G = 1.0 V. (f) V_G = 2.0 V. (g) V_G = 3.0 V. (h) Schematic diagram of Si/Al_2O_3/CVD grown MoS_2.

Figure S4 shows the light stimulated potentiation and drain current retention of CVD grown monolayer MoS_2 on Al_2O_3 gate at different gate voltages. At negative gate voltages, device potentiates with application of each light pulse and retains a huge part of drain current after removing the light source. Device shows maximum retention at V_G = -1.0 V. Therefore, device characteristics does not change for replacing SiO_2 gate with Al_2O_3 gate dielectric.

S5. Drain current retention of exfoliated MoS_2 on SiO_2 gate.

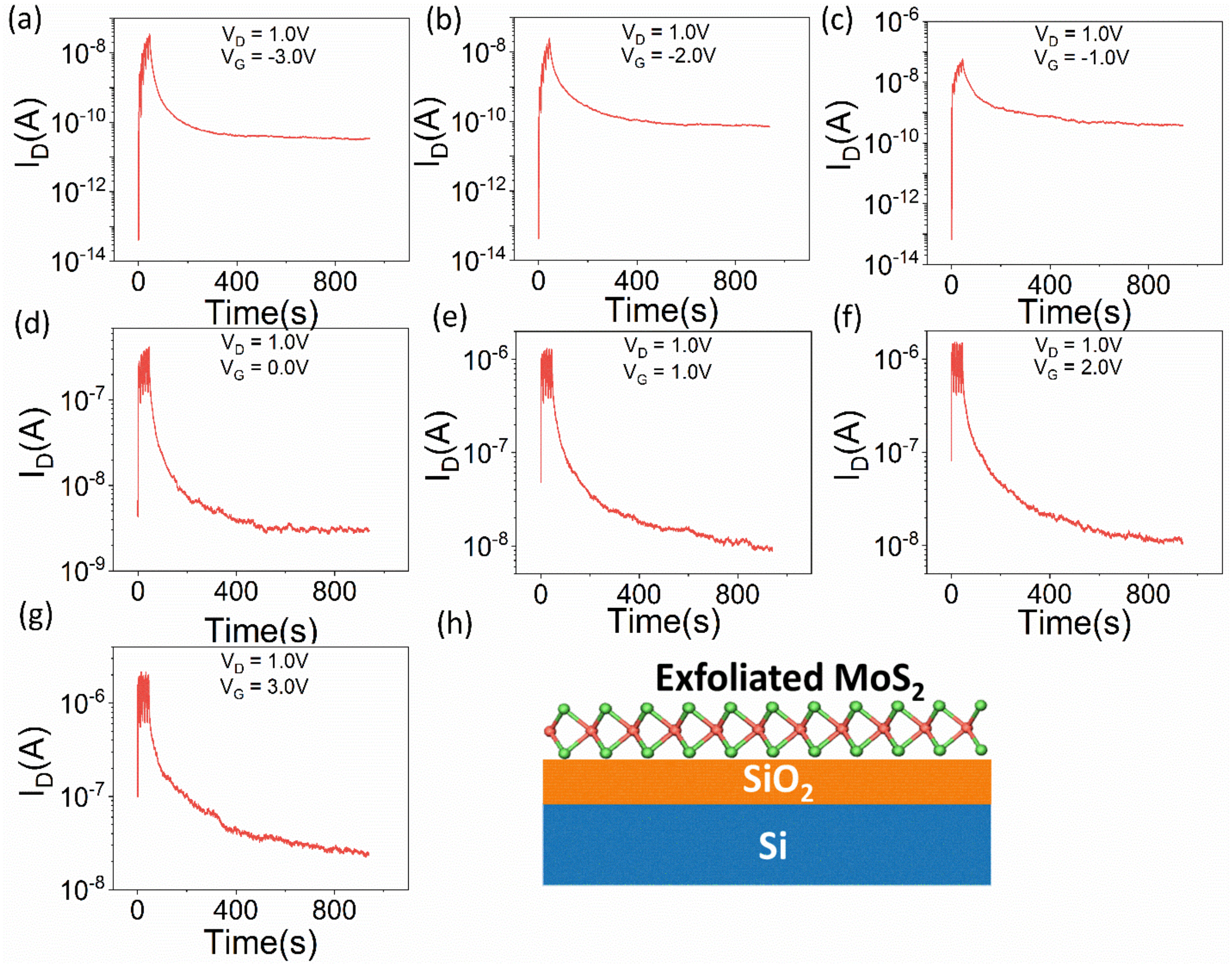

Figure S5. Light stimulated potentiation and drain current retention of exfoliated MoS_2 on SiO_2 gate at (a) V_G = -3.0 V. (b) V_G = -2.0 V. (c) V_G = -1.0 V. (d) V_G = 0.0 V. (e) V_G = 1.0 V. (f) V_G = 2.0 V. (g) V_G = 3.0 V. (h) Schematic diagram of Si/SiO_2/Exfoliated MoS_2.

Figure S5 shows the light stimulated potentiation and drain current retention of exfoliated MoS_2 on SiO_2 gate at different gate voltages. At negative gate voltages, device potentiates with application of each light pulse and retains a huge part of drain current after removing the light source. Device shows maximum retention at V_G = -1.0 V. Therefore, device characteristics does not change for replacing the CVD grown MoS_2 with exfoliated MoS_2.

S6. I_D - V_G, I_D – V_D and drain current retention of exfoliated WSe_2 on SiO_2 gate

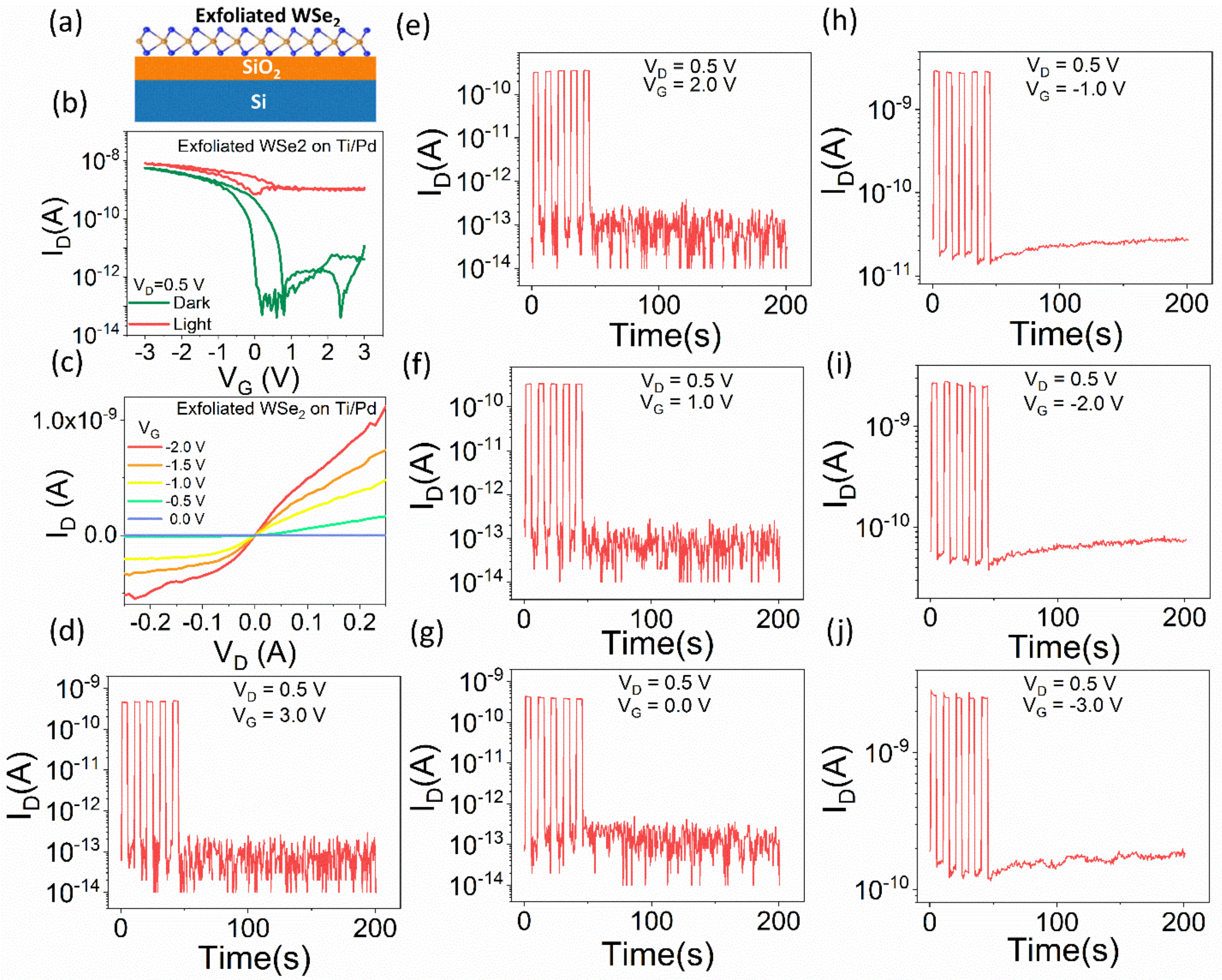

Figure S6. Exfoliated WSe_2 FET on SiO_2 gate: (a) Schematic diagram (b) I_D - V_G at V_D = 0.5 V in dark and under light illumination, (c) I_D - V_D at different V_G in dark. Light stimulated potentiation and zero drain current retention of exfoliated WSe_2 on SiO_2 gate at (d) V_G = 3.0 V. (e) V_G = 2.0 V. (f) V_G = 1.0 V. (g) V_G = 0.0 V. (h) V_G = -1.0 V. (i) V_G = -2.0 V. (j) V_G = -3.0 V.

Figure S6 shows the exfoliated WSe_2 FET on SiO_2 gate (a) Schematic diagram (b) I_D - V_G at V_D = 0.5 V in dark and under light illumination, (c) I_D - V_D at different V_G in dark. Light stimulated potentiation and drain current retention of exfoliated WSe_2 on SiO_2 gate at different gate voltages. Device did not show any retention of drain current for any of the applied gate voltages.

Therefore, WSe_2/MoS_2 interface is not capable of trapping the photogenerated holes. So, the photogenerated carriers recombine as soon as the light source is removed.

S7. Paired pulse facilitation measurement.

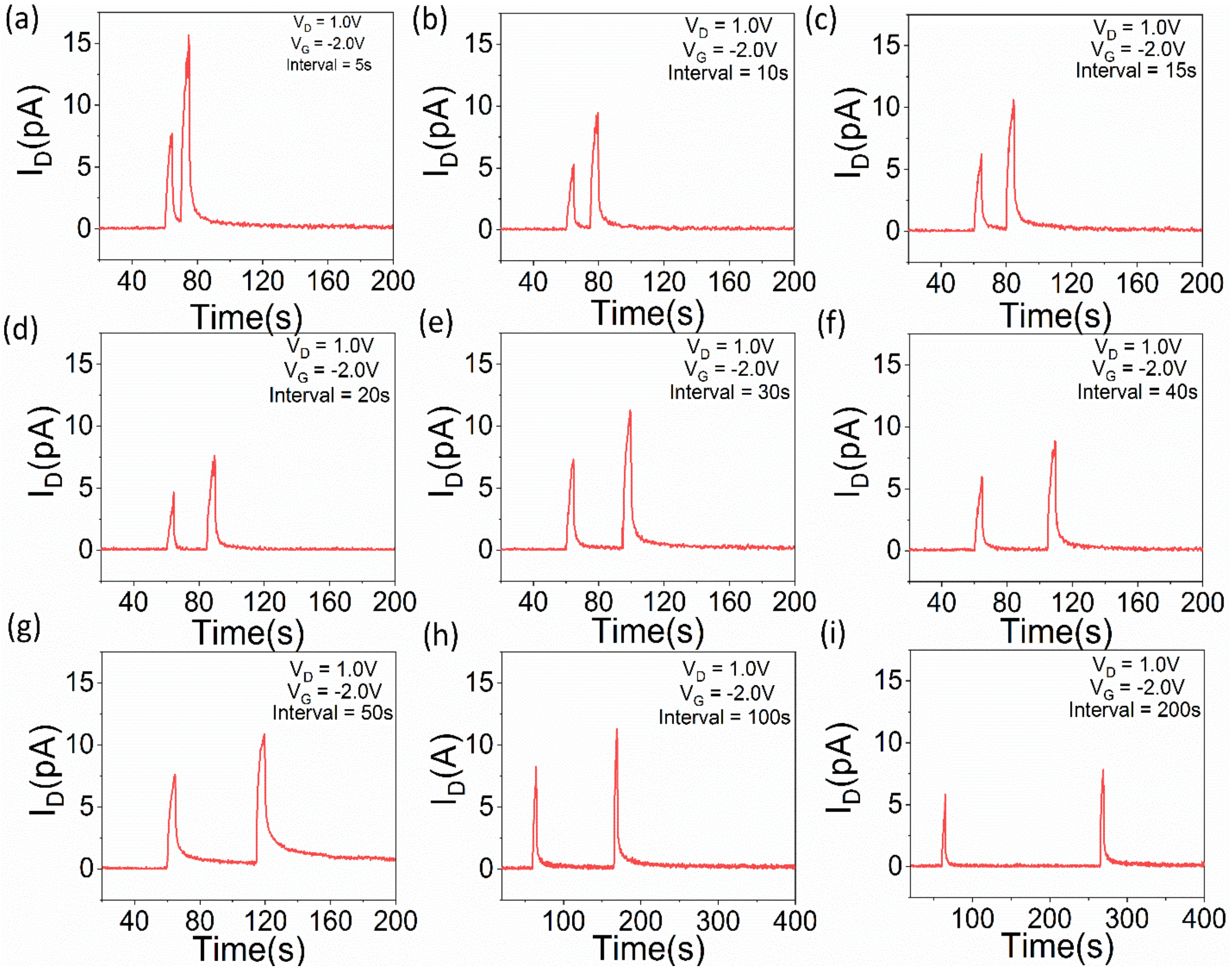

Figure S7. Paired pulse facilitation measurement at V_D = 1.0 V and V_G = -2.0 V for the time interval (a) 5 s, (b) 10 s, (c) 15 s, (d) 20 s, (e) 30 s, (f) 40 s, (g) 50 s, (h) 100 s, (i) 200 s between two successive light pulses.

Figure S7 shows the paired pulse facilitation measurement at V_D = 1.0 V and V_G = -2.0 V for different time intervals. Two successive light pulses were applied to the device. With increasing

time difference between the two applied pulses, the drain current amplitude after second pulse decreased gradually. Maximum drain current after second light pulse was recorded for minimum time interval between the two applied pulses.

S8. Spike time dependent plasticity measurement.

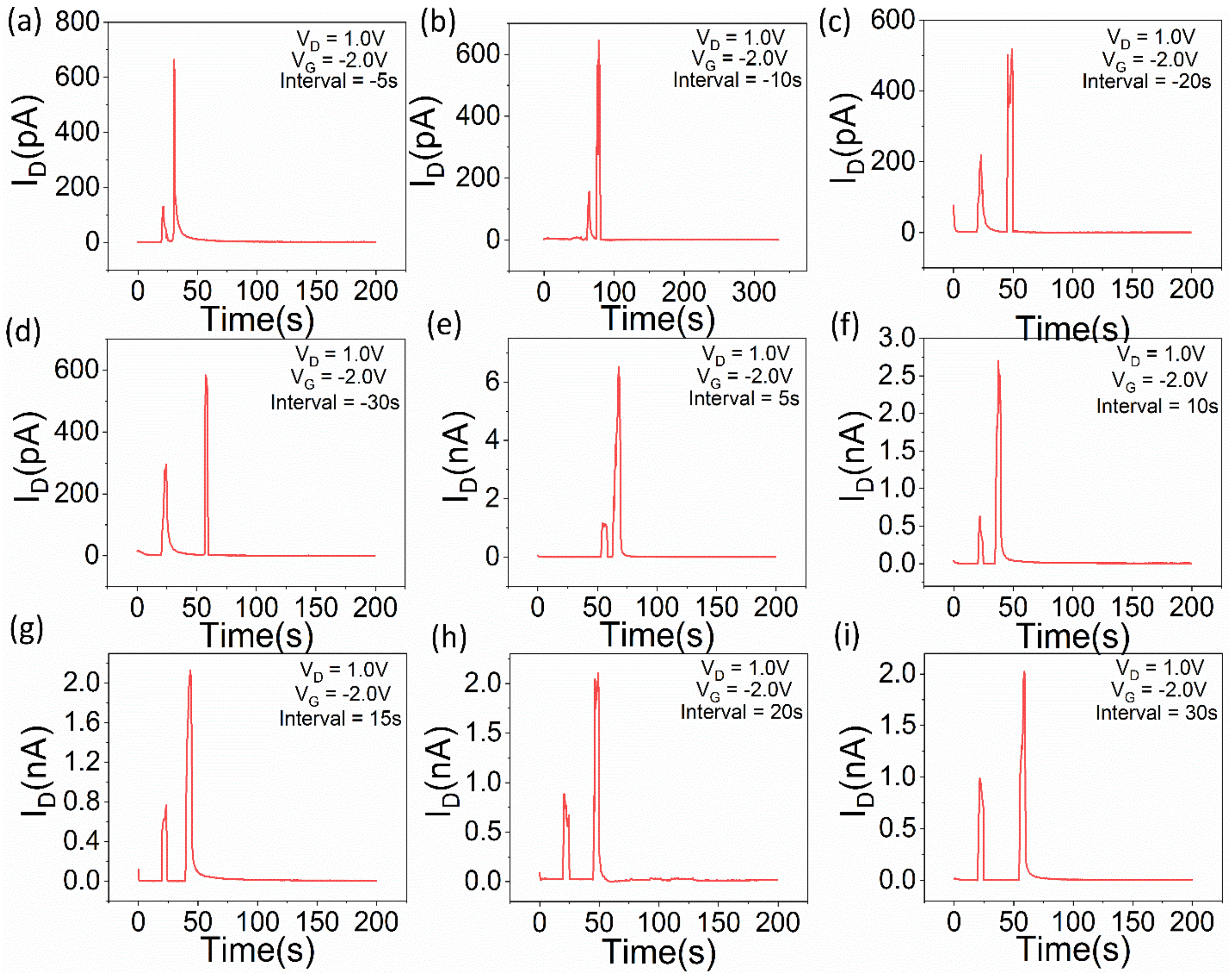

Figure S8. Spike time dependent plasticity measurement at V_D = 1.0 V and V_G = -2.0 V for the time interval (a) -5 s, (b) -10 s, (c) -20 s, (d) -30 s, (e) 5 s, (f) 10 s, (g) 15 s, (h) 20 s, (i) 30 s between two successive light pulses.

Figure S8 shows the spike time dependent plasticity measurement for different time intervals between two applied light pulses. Two light pulses were applied to two electrically shorted devices separately with a varied time interval at V_G = -2.0 V and V_D = 1.0 V. With increasing time interval between two applied light pulses, the drain current amplitude after second pulse decreased gradually. When first light pulse was applied on presynaptic device, the time interval was considered positive and when first light pulse was applied on postsynaptic device, the time interval was considered negative.
